# Bacteriophages as Potential Tools for Detection and Control of *Salmonella* spp. in Food Systems

**DOI:** 10.3390/microorganisms7110570

**Published:** 2019-11-17

**Authors:** Shuai Wei, Ramachandran Chelliah, Momna Rubab, Deog-Hwan Oh, Md Jalal Uddin, Juhee Ahn

**Affiliations:** 1 Department of Medical Biomaterials Engineering, College of Biomedical Science, Kangwon National University, Chuncheon, Gangwon 24341, Korea; weishuaiws@126.com (S.W.); jalalmbe@kangwon.ac.kr (M.J.U.); 2 Guangdong Provincial Key Laboratory of Aquatic Product Processing and Safety, College of Food Science and Technology, Guangdong Ocean University, Zhanjiang 524088, China; 3 Department of Food Science and Biotechnology, College of Agriculture and Life Science, Kangwon National University, Chuncheon, Gangwon 24341, Korea; ramachandran865@gmail.com (R.C.); rubab.momna@gmail.com (M.R.); deoghwa@kangwon.ac.kr (D.-H.O.)

**Keywords:** bacteriophage, phage detection, phage control, *Salmonella*, food system

## Abstract

The global problem of antibiotic resistance in bacteria is quickly developing in most antibiotics used in hospitals and livestock. Recently, the infections with multi-drug resistant (MDR) bacteria become a major cause of death worldwide. Current antibiotics are not very effective in treating MDR *Salmonella* infections, which have become a public health threat. Therefore, novel approaches are needed to rapidly detect and effectively control antibiotic-resistant pathogens. Bacteriophages (phages) have seen renewed attention for satisfying those requirements due to their host-specific properties. Therefore, this review aims to discuss the possibility of using phages as a detection tool for recognizing bacterial cell surface receptors and an alternative approach for controlling antibiotic-resistant pathogens in food systems.

## 1. Introduction

*Salmonella*, a Gram-negative rod-shaped bacilli bacterium, is the most common foodborne pathogen and is known as one of the four key global causes of diarrheal diseases, according to the World Health Organization (WHO). As estimated by the Centers for Disease Control and Prevention, 23,000 hospitalizations and 450 deaths have been caused by *Salmonella* every year in the United States. Among the 1.2 million illnesses caused by *Salmonella*, contaminated food caused 1 million illnesses [1,2]. Contaminated meat, poultry, eggs, and milk are the main reservoirs for *Salmonella* infections [3]. Some direct contact with infected animals, blood, urine, and feces may also cause the problem to human health [4]. Antibiotic use has been increased for controlling the bacteria in animals or increasing food production, which has accelerated the emergence of antimicrobial-resistant bacteria [5,6,7]. The antibiotic-resistant bacteria are likely to contaminate in food products throughout the handling or other production stages [8,9,10]. Fruit and vegetable products are susceptible to cross-contamination during harvest and post-harvest periods [11,12,13]. Salmonellosis caused by *Salmonella* is one of the serious infections that is difficult to treat due to the reduced antibiotic activities that become less effective [14]. Thus, novel antibiotics or alternative methods are required to control the antibiotic-resistant bacteria.

Bacteriophages (phages) are predominant in nature, defined as viruses that can infect bacteria alone [15,16]. In the 1920s, Frederick Twort first observed the glassy and transparent colonies in micrococci cultures, which could cause an acute infectious disease in 1915, but the term “phage” was neither correctly defined nor clearly understood at this time [17]. Two years later, Félix d’Herelle isolated a microbe that showed an antagonistic effect against *Shiga* bacillus and first described this microbe as an obligate bacteriophage with a high specificity to the host and no pathogenic effects [18].

**Figure 1:**
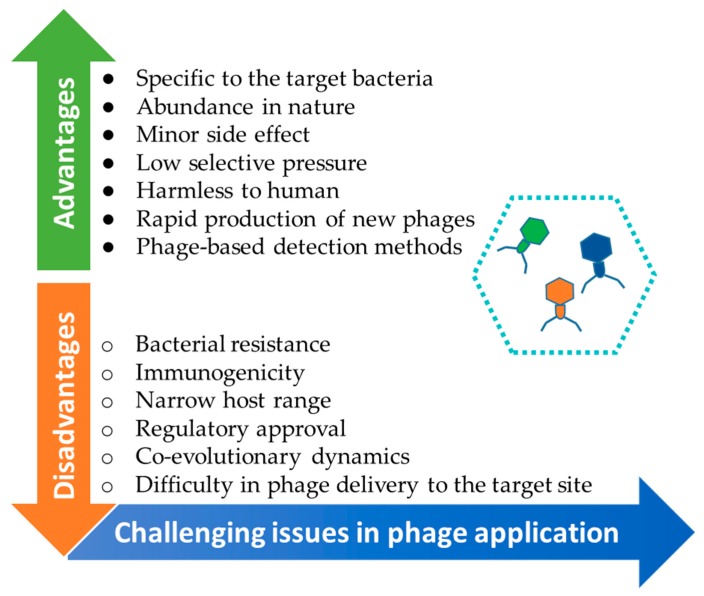
Advantages and disadvantages of using bacteriophages for the treatment of *Salmonella* [19].

However, the interest in phages decreased because of the lack of proper quality controls and reproducible results in the western countries [21,22,23]. Another important reason was the discovery of antibiotics, which were used as the most powerful reagents for controlling bacterial infections. Ironically, the frequent use of antibiotics has resulted in the development of the multidrug-resistance or superbugs [24,25,26]. Figure 1 shows the advantages and disadvantages of bacteriophage applications. A phage as a biorecognition agent provides many advantages for rapid bacterial sensing [27], including target specificity [28], release of high titer phages, tolerance to environmental stresses [29], safe handling, and effectiveness against live bacteria [30]. The phage-based biosensors include the combination of whole phages or phage-constituents, which can be converted to electrical, colorimetric, fluorescent, or luminescent signals. Phages are thus shown as cheap, fast, sensitive, selective, and specific tools for detecting bacteria [31]. From a therapeutic viewpoint, phage therapy provides many benefits over chemotherapy, since phages are active against antibiotic-resistant bacteria and no side effect occurs during phage treatment [32].

With more scientifically rigorous approaches in the recent years, more researchers have paid attention toward the bacteriophages as a promising tool to treat the pathogens [33]. Bacteriophages also show additional advantages, including a high specificity to the host, an ability to differentiate alive and dead cells, and the most abundant biological entity in nature, which draws renewed attention to the detection and control of antibiotic-resistant pathogens [34,35,36]. The effectiveness of phage applications in treating pathogenic bacteria is influenced by several factors, such as the multiplicity of infection (MOI), treatment mode, environmental conditions, phage neutralization, and target bacteria. The bacteriophage survival an adverse environment is also a desired characteristic for therapeutic use. Currently, the commercial products based on bacteriophages targeting *Escherichia coli* O157:H7 [37,38,39], *Listeria monocytogenes* [40,41,42], *Salmonella* spp. [43,44], and *Shigella* spp. [45,46,47] are available in the market. Furthermore, application trials in foods are also performed, which would help enhance the food safety. The lytic activities of specific bacteriophages against *Salmonella* and other pathogenic bacteria are compared in Table 1 and Table 2. In this review, the detection methods and biocontrol applications based on bacteriophages targeting *Salmonella* are summarized and discussed in detail.

## 2. Phage Plaque Assay as A Traditional Method for *Salmonella* Detection

**Figure 2:**
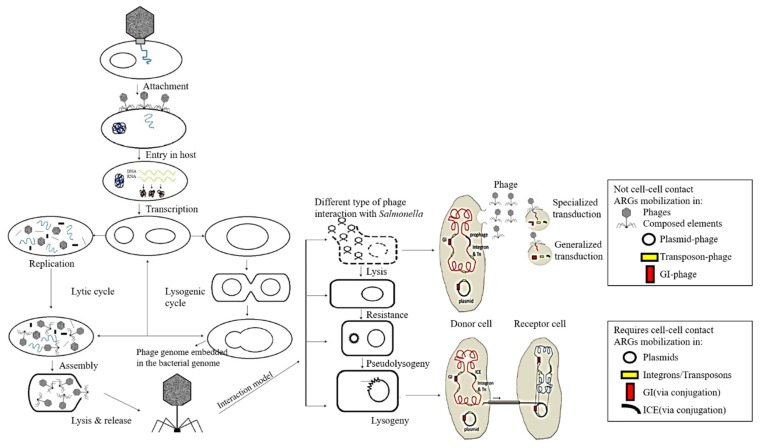
Life cycles of bacteriophages. (Copyright obtained from Kakasis et al., 2019; de Jonge et al., 2019, [57,58].)

The traditional agar overlay method is a very useful technique for studying bacteriophage, including identification, enumeration, purification, and isolation of phage mutants. This method is based on lysis plaques, which are formed on lawns of the host bacteria, immobilized in the top soft agar. After incubation, a series of reaction events, such as phage infection, multiplication, and liberation, occur in the host [49,50]. The soft agar overlay method is a common titer assay, which was introduced by Mark Adams [51]. The plaque assay has been improved based on several modifications, such as the addition of effective supplements to modify growth media and agar composition and the plate spreading method, which can enhance the visibility of the plaques [49,52,53]. However, a long time (more than 24 h) is required for obtaining detection results and it is also labor intensive, which may not meet the demands of rapid pathogen detection. Thus, many researchers devote their efforts to developing reliable and rapid detection methods. Figure 2 shows two different life cycles of bacteriophages and lytic and lysogenic cycles. The active virulence phages can produce progeny phages that burst out of the host cell through the lytic cycle, while the lysogenic cycle involves the integration of the temperate phage genome into the host chromosome, which can remain in an inactive state, known as prophage [54]. The distinct life cycles of bacteriophages are used for designing novel diagnostic tools, including reporter bacteriophages [55] and phage display technologies [56].

## 3. Rapid Detection Methods Associated with Bacteriophage for *Salmonella*

With the growing need of food safety, several detection methods for targeting *Salmonella* were developed by combining bacteriophages, including molecular based real-time PCR [59,60], immunomagnetic separation based on fluorescence immunoassays [61,62], enzyme-linked immunosorbent assays (ELISAs) [59], matrix-assisted laser desorption/ionization–time-of-flight mass spectrometry (MALDI–TOF MS) [63], and genetically engineered reporter phage [64].

### 3.1. qPCR-Based Detection

Bacteriophages have been used for the detection of foodborne pathogens based on their specificity towards bacteria and amplification ability, which is like the “enrichment” procedure and shortens the detection time [36]. In this approach, qPCR is used to directly amplify and detect the nucleic acids of progeny bacteriophage after propagation. The bacteriophages coupled with qPCR were used for the detection of *Ralstonia solanacearum*, *Escherichia coli* O157:H7, *Mycobacterium avium*, and *Acinetobacter baumannii* [60,65,66,67]. The amplification of *Salmonella* phage B_SenS_PVP-SE2 combined with qPCR was then developed for the detection of viable *Salmonella* Enteritidis in chicken samples [68]. The proposed method detected a low concentration level of viable *S.* Enteritidis (8 colony-forming unit (CFU)/g) in chicken samples within 10 h, which saved much time when compared to the culture-based method and also enhanced sensitivity, specificity, and accuracy. Thus, this protocol can be used in the food industry for self-monitoring, which successfully completed a “same-day” detection within 10 h.

### 3.2. Immunomagnetic-Based Detection

A novel assay composed of immunomagnetic separation (IMS) and amplification of *Salmonella* bacteriophage SJ2 was developed and optimized for the detection of *Salmonella enterica* serovar Enteritidis [62]. In the IMS procedure, Dynabeads^®^ anti-*Salmonella* was used for capturing and concentrating *Salmonella*. Bacteriophage SJ2 was then added, and the mixture was incubated for attachment and amplification. The final sample was detected using fluorescence or optical density measurements. This assay showed a detection limit of less than 10^4^ CFU/mL with a short time (4.0–4.5 h). However, the pre-enrichment process was required in food samples. When this technique was applied to contaminated food samples, including skimmed milk, chicken, and beef, at an average of 3 CFU/25 g, *S*. Enteritidis could be detected within 20 h, including a pre-enrichment time of 16 h [62].

### 3.3. Enzyme-Linked Immunosorbent Assay

The commercial ELISA kits have been applied for detecting *Salmonella* in poultry, seafood, milk, and meat [69,70,71,72]. ELISA detects the protein in a liquid form using antibodies against the target samples. Instead of using antibodies, bacteriophages can be used in ELISA for detecting bacteria [73]. The ELISA procedure works by replacing antibodies with bacteriophages and was applied for the detection of *S. enterica* and *E. coli* [59]. The modified ELISA showed a detection limit up to 10^6^ cells/mL, which is comparable with other ELISA methods. Thus, bacteriophages in ELISA can be an alternative way to detect pathogenic bacteria without specific antibodies. Since phages are highly abundant in nature, this assay becomes cheap compared with using specific antibodies.

### 3.4. Matrix-Assisted Laser Desorption/Ionization–Time-of-Flight Mass Spectrometry

MALDI-TOF MS is now becoming a most common method for bacterial identification, differentiating from the advantages of high throughput and rapidity and excepting a high cost of the initial installment [74,75]. MALDI-TOF MS has been applied for screening, identification, and detection of foodborne bacteria, which can enhance food safety. The MALDI-TOF MS-based detection method depends on progeny bacteriophage proteins or peptides. In this method, progeny bacteriophage proteins are applied to the test plate with a UV-absorbing matrix and then ablated by a laser. After 60–120 min of bacteriophage amplification, samples were used for analysis by MALDI-TOF MS. The parameters of MALDI-TOF MS, including matrix preparation, sample preparation, acid added to the matrix, growth medium, and setting parameters, were optimized and a standard protocol was set for the identification of *Salmonella* subspecies, and consequently the classification results were comparable to DNA sequence-based methods [76]. A whole-cell MALDI-TOF MS for rapid prescreening of *S*. *enterica* subspecies *Enterica,* isolates based on specific biomarker ions, rather than antigenic determinants that could reduce the sample numbers for subsequent serotyping analysis [77]. The MALDI-TOF MS combined with selective enrichment broth was developed for the identification of *Salmonella* sp. in clinical stool samples. The discrimination of bacteria species was mainly based on the comparison of peaks of peptides and small proteins with the reference database [78]. Strains of *Salmonella* and *E. coli* were simultaneously detected based on the characteristics of proteins by using two phages, MS2 and MPSS-1, respectively, [63]. The simultaneous detection of two bacteria, using MALDI-TOF MS coupled with bacteriophage amplification, provides the possibility of three or more target detections, which may require the specific bacteriophage biomarkers.

### 3.5. Genetically-Engineered Phages

Recent advances in genetically-engineered bacteriophages have been created as a powerful tool for the monitoring and detecting of bacterial pathogens. This novel technique is useful for detecting bacteria from contaminated foods with high selectivity and sensitivity [64,79]. Reporter bacteriophages are genetically modified bacteriophages that have a reporter gene inserted into their genomes, such as *lux*, *gpf*, and *lacZ*, which are activated by the interaction between bacteriophages and target bacteria [64,80]. The expression of the reporter gene upon infection emits a detectable signal, indicating the presence of target bacteria. The main advantage of using a reporter bacteriophage is the higher specificity for detecting viable host bacteria. To date, most commonly used reporter bacteriophages are associated with the formation of bioluminescence luciferase protein, which emits light in the presence of aldehyde substrates [81]. Several bioluminescent reporter bacteriophage systems have been designed using the lux operon, *luxCDABE*. The LuxCDE proteins encode a fatty acid reductase complex, including reductase, synthetase, and transferase, responsible for providing the aldehyde as a substrate, and the LuxAB encodes luciferase α- and β-subunits, which feed the bioluminescent reaction [82,83]. Reporter bacteriophages containing the *luxAB* gene were the first bioluminescent for the detection of *Salmonella* strains [84]. The *luxAB* (P22 luxAB) reporter gene without the luxCDE was used to detect *S*. Enteritidis up to 63 CFU/g in whole eggs [84]. The advantage of the *luxAB* reporter system is that it can avoid the toxicity and noise signals by emitting a physical signal. However, this system needs the specific substrates for bacterial detections [82]. To avoid this inconvenience, a complete set of *luxABCDE* operons was constructed for *Salmonella* Typhimurium detection in different food matrices [83]. The reporter bacteriophage system could detect *Salmonella* up to 37 CFU/g in sliced pork, 22 CFU/g in iceberg lettuce, and 20 CFU/mL in pure culture. These reporter bacteriophages could be useful for diagnostics and rapid detection of *Salmonella* spp. in different food samples with no substrates and with the reporter host required.

Apart from the luciferase-based reporter bacteriophage systems, green fluorescent protein (GFP) and β-galactosidase have also been used for the detection of foodborne pathogens. The GFP gene is originated from *Aequorea victoria* and has many advantages, including high stability and low toxicity [85,86]. The GFP-labeled PP01 bacteriophage (PP01-GFP) system was applied on the surface of *E. coli* O157:H7, which can emit a fluorescent signal at an MOI of 1000 at 4 °C [87]. Although the specificity and host range are well defined, this system is not widely used in food because of the interference of food components. The *lacZ* gene encoding β-galactosidase can catalyze the hydrolysis of β-galactosides. The *lacZ*-based reporter bacteriophage needs various substrates, such as colorimetric, fluorescent, or luminescent substrates, which emit signals to detect bacteria [36,88]. The detection limits of this system were up to 10^3^ CFU/100 cm^2^ for the colorimetric method and 10 CFU/100 cm^2^ for luminescence in beef slice samples [88]. The use of additional substrates can be a drawback of this system as it only allows single time point measurements. Although this reporter bacteriophage system can detect viable *Salmonella* at high specificity and a low detection limit, the construction of new reporter bacteriophages is still difficult. Therefore, further studies are needed for the development of reporter bacteriophage systems, which can detect *Salmonella* spp. in various food matrices.

## 4. Bacteriophage-Based Biosensors for Detecting *Salmonella*

Over the last few decades, biosensors have been developed as a novel analytical platform for pathogen detection [89,90]. A classical biosensor can be defined as an analytical device that measures biological responses by incorporating bioreceptors (antibodies, enzymes, cells, aptamers, bacteriophage, and organelle) with physical transducers and electrochemical (amperometric, impedimetric, and potentiometric), optical (surface plasmon resonance, surface-enhanced resonance spectroscopy, and fluorescence), and mass-based receptors (magnetoelastic and piezoelectric). The continuous efforts have been successfully developed on bacteriophage-based biosensors for the detection of *Salmonella* in food samples. The immobilization of bacteriophage receptors on the sensor surface is crucial to develop bacteriophage-based biosensors. The immobilization steps include physical adsorption, covalent attachment, and genetic modification of receptors [91,92].

**Figure 3:**
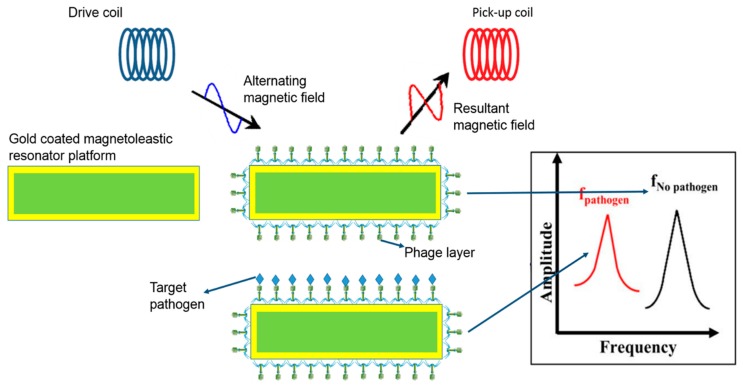
Schematic figure illustrating the working principle of ME biosensors. [93].

Appendix A summarizes different bacteriophage-based biosensors for the detection of *Salmonella*. The first transducers of bacteriophage biosensors for *Salmonella* detection are mass-based transducers and magnetoelastic assays (ME), and Figure 3 shows a schematic illustration for the principle of ME biosensors for detection of target analytes [93].

ME-based detection methods are the most prominent type of biosensors due to their easy and cheap fabrication, composing of amorphous ferromagnetic ribbon that contracts and expands when exposed to the external magnetic field and generates magnetic fluxes by binding targets to the sensor surface. The ME biosensor was employed as a transduction platform in bacteriophage biosensors for the detection of *Salmonella* (Table A1). ME biosensors were developed by using filamentous bacteriophage specific for *S.* Typhimurium. The bacteriophages were immobilized by a physical adsorption method for *S.* Typhimurium detection by the changes in the resonance frequency of the sensor [93]. The numbers of *S.* Typhimurium in fresh tomato surfaces were quantified using the ME biosensor detection method by the immobilization of E2 bacteriophages on the sensor surface [94]. The tomato surfaces contaminated with *S.* Typhimurium were measured using a resonance frequency with a detection limit of 10^3^ CFU/mL. The results show that E2-bacteriophage-based biosensors could detect *Salmonella* directly on the surface of tomatoes. The same principle for detection of *S.* Typhimurium in spinach leaves showed a similar detection limit as low as 10^2^ CFU/mL [94].

Other bacteriophage-based biosensor detection systems have been developed using an acoustic wave piezoelectric biosensor combined with filamentous bacteriophages [95]. The detection limit was 10^2^ CFU/mL for *S.* Typhimurium by measuring the changes in resonance frequency as a consequence of binding bacteria to the bacteriophage. In addition, the recombinant prophage coupled with a flow cytometer and specific fluorescence filter was used for sensitive and specific detection of *Salmonella* with a detection limit of 10 CFU/mL [86]. A surface enhanced Raman scattering (SERS) by conjugating bacteriophage tail spike proteins to silica-encapsulated Raman reporter-embedded nanoprobes could detect single *Salmonella* cells [96]. Therefore, the use of bacteriophage as a bioreceptor in biosensors can contribute to the development of desirable detection tools for *Salmonella* in food samples. The stability, low cost, environment-friendly production, and genetic modification provide benefits for biosensor development. For the successful development of biosensors, the immobilization of phage onto the biosensor surface plays an important role. The genetically modified phages provide effective immobilization by introducing the functional ligands on their heads. In addition, the ability to manipulate the genetic material provides the possibility of creating novel recognition systems for biosensor applications, such as expanding the host range of phages by manipulating the receptor-binding protein [27]. However, further work should focus on detecting *Salmonella* in complex food matrices for extending the range of application of bacteriophage-based diagnostic tools from the laboratory to clinical diagnosis, environmental monitoring, and further food analysis in the near future.

## 5. Bacteriophage-Based Tool for *Salmonella* Control

Bacteriophages have been used for controlling bacterial infections based on their specificity to the host bacteria [35,97,98,99,100,101,102,103]. Bacteriophages kept stable in thermal conditions from 30 to 60 °C and pH ranges from 3 to 13 can suggest the possibility of using bacteriophages in variable conditions. Recently, bacteriophages as a biocontrol tool have gained great attention and are recognized as an alternative for antibiotics [104,105,106]. *Listeria* bacteriophages were approved by the Food and Drug Administration (FDA) and the United States Department of Agriculture (USDA) in all food products, which were granted as generally recognized as safe (GRAS) [107]. Appendix B summarizes the applications of bacteriophage or bacteriophage-based treatments as biocontrol tools for *Salmonella*.

Bacteriophage control technique has been applied for *Salmonella* in vivo and food samples. When lytic bacteriophages was applied to the chicken skin contaminated with *S. enterica* serovar Enteritidis, less than one log reduction was obtained at the MOI of 1 and no viable bacteria were observed at the MOI of 10^5^ [108]. A new virulent bacteriophage, F01-E2, was isolated for controlling *S.* Typhimurium [109]. F01 belongs to *Myoviridae* with a double-stranded deoxyribonucleic acid dsDNA genome of 86.2 kb and a broad host range [110,111].

Five log reductions were obtained for turkey deli meat and chocolate milk at 15 °C, and three log reductions were observed for hot dogs and seafood, implying that bacteriophage immobilized on the food surfaces were affected by the structure and chemical composition of the foods [112]. *Salmonella* Enteritidis bacteriophage SE07 showed a potential against *S.* Enteritidis in both solid and liquid food [113]. The isolated SE07 belongs to *Podoviridae* and is stable from 28 °C to 65 °C and pH 4 to 11. As shown in Table 3, two log reductions were obtained for the different food matrices after 48 h incubation at 4 °C. Additionally, the bacteriophage *ΦCJ07* was applied for controlling *S.* Enteritidis in chicken [114].

Because *Salmonella* in contaminated, chickens can survive under the acidic conditions in the digestion system. Bacteriophage *ΦCJ07* was added as a feed additive, which was effective against *Salmonella* by protection from other ingested feed constituents [115]. The bacteriophage *ΦCJ07* isolated from the sewage effluent showed a lytic activity against most *Salmonella* spp., including *S.* Enteritidis, *S.* Typhimurium, *Salmonella* Gallinarum, *Salmonella* Pullorum, *Salmonella* Choleraesuis, and *Salmonella* Derby. Further evaluations in vivo also demonstrated the good performance of phage *ΦCJ07* in reducing both *S.* Enteritidis colonization and environment contamination levels. This provides a promising alternative of bacteriophage for preventing and controlling *S.* Enteritidis infections and reducing the incidence of *Salmonella* food poisoning.

### 5.1. Phage Cocktails

Despite the advantages of bacteriophages, bacteria can become resistance to bacteriophages through surface modification, superinfection exclusion, restriction modification, abortive infection, and clustered regularly interspaced short palindromic repeatsCRISPR-associated 9 (Cas9) systems [117,118]. Therefore, phage cocktails with different host specificities have been of great interest and are more practical for expanding the bacteriophage application, since the combined bacteriophage cocktails can reduce the development of bacteriophage-resistant mutants.

A mixture of two bacteriophages was used for controlling *Salmonella* in sprout seeds [119]. Bacteriophage A belongs to the *Myoviridae* family, while bacteriophage B is a member of the *Siphoviridae* family. The reductions of *S.* Typhimurium, *S.* Enteritidis, and *Salmonella* Montevideo were noticeable at the bacteriophage mixture of A and B in broccoli seeds compared to single bacteriophage treatment. The isolated bacteriophages effectively reduced the numbers of *S.* Typhimurium and *S.* Enteritidis in chickens [120]. The mixture of three phages, UAB_Phi20, Phi78, and Phi87, showed higher lytic activity than that obtained by any of the three phages alone, while the phage cocktail lysed *Salmonella* Virchow, *Salmonella* Hadar, *Salmonella* Infantis, *S.* Typhimurium, and *S.* Enteritidis, showing a broad spectrum lytic capability. The bacteriophage cocktail was applied in different food systems [121]. Significant reductions of *S.* Typhimurium and *S.* Enteritidis were observed for different food matrices, including pig skin, chicken breasts, and lettuce. Recently, a phage cocktail (BSPM4, BSP101, and BSP22A) based on targeting different cell surface receptors, including flagella, O-antigen, and BtuB, has been developed for the inhibition of *Salmonella* Typhimurium from fresh produce foods [122]. The multiple receptor-targeting bacteriophage cocktail can reduce *Salmonella* by up to 4.7–5.5 log CFU/cm^2^ in iceberg lettuce and 4.8–5.8 log CFU/cm^2^ in cucumber after 12 h incubation at 25 °C, without the development of bacteriophage resistance [122]. At present, the commercial bacteriophage cocktail has been applied for controlling *Salmonella* in poultry products [123].

### 5.2. Phage Endolysins

Bacteriophage endolysins have been used as a novel biocontrol agent and natural food preservatives over the past decades. The endolysins are peptidoglycan hydrolases that can lyse host cells after phage replication and propagation. The endolysins are mainly active against Gram-positive bacteria, which do not contain an outer membrane [36,124]. The outer membrane of Gram-negative bacteria can prevent contact between free endolysins and peptidoglycan. However, some *Salmonella* bacteriophage endolysins can bypass the outer membrane barriers when combined with different outer membrane permeabilizers, such as ethylene diamine tetra-acetic acid (EDTA), citrate, and malate [114,125,126]. The bactericidal activity of a *Salmonella* phage endolysin (Lys68) combined with organic acids was increased against Gram-negative bacteria [127]. A *Salmonella* bacteriophage endolysin, Gp110, has currently proved to show enzymatic activity [128]. In addition, bacteriophage endolysins have also been engineered to increase the bactericidal effect against Gram-negative bacteria. The modified endolysin combined with lipopolysaccharide (LPS)-destabilizing peptides showed promising results against *Pseudomonas aeruginosa*, showing more than 5 log CFU/mL reduction. However, there are still some limited effects against *Salmonella* Typhimurium (<1 log CFU/mL reduction) [129]. Although many bacteriophage endolysins have been introduced and characterized, further optimization is still needed to increase the host specificity and lytic activity. The genetic engineering endolysin can be one of the useful approaches for satisfying these requirements.

### 5.3. Phage Control Combined with Other Preservatives

The hurdle concept (or barrier technology) is applied to foods to enhance the microbiological safety and quality. Many preservative methods are employed, together with other barriers to effectively control microbial contamination in food [130]. Many studies have demonstrated bacteriophages as alternative antimicrobials to control bacteria. Several *Salmonella* bacteriophages, such as Salmonelex™, SalmoFresh^TM^, and SalmoPro^TM^, have been approved as GRAS by the United States Food and Drug Administration (US FDA) and the US Department of Agriculture’s Food Safety and Inspection Service (USDA-FSIS) [131,132]. The combinations of bacteriophages and antimicrobials or sequential applications showed an effective biocontrol ability against the target bacteria [38,133]. SalmoFresh^TM^ bacteriophages combined with cetylpyridinium chloride (CPC) or lauric arginate (LAE) showed more than 5 log reductions against *Salmonella* spp. in chicken products [133,134,135,136]. However, an in vivo test on chicken breast fillets showed that a lower number of *Salmonella* (0.5 to 1.3 log CFU/g) was reduced by the combinations of bacteriophages with CPC or LAE, which may be attributed to the complex matrix of the meat components [137]. Sequential treatment of chlorine, CPC, LAE, or peracetic acid (PAA) with concentrations of 50 and 400 ppm, respectively, followed by phage spray, were carried out to evaluate the hurdle effect of *Salmonella* on chicken skin. The high reductions of 1.7 to 2.2 and 2.2 to 2.5 log CFU/cm^2^ were obtained with an immersion in 50 and 400 ppm of PAA, followed by phage spray, which may be used in industries for the reduction of *Salmonella* contamination in cut meat. With growing interest of the combinations of bacteriophages and antimicrobials, further studies are needed to evaluate the inhibitory effect of antimicrobials combined with bacteriophages, the potential synergistic effect of the combination, and the mode of phage application, such as immersion and spraying [38,138,139,140].

## 6. Conclusions

Notably, research related to bacteriophages and their promising applications has increased in recent decades due to frequent outbreaks and the emergence of antibiotic-resistant bacteria. The effective detection and biocontrol of *Salmonella*, based on the potential bacteriophages, are of importance to reduce the incidence of *Salmonella* and ensure the food safety. Since many studies have been performed in the laboratory with well-controlled conditions, bacteriophages showed a significant effect on the inhibition of bacteria both in vivo and in vitro. Novel hurdle technology-coupled phages with antimicrobials, UV, or antagonistic bacteria are of interest to find synergistic effects against pathogens, which provide potential effective ways to be used in industries for control pathogens and alleviate the risk of pathogen contaminations in foods. 

## Figures and Tables

**Table 1:** Lytic spectrum of bacteriophages against *Salmonella* and other foodborne pathogens.

Microorganism	Bacteriophage ^1^				
	7	16	18	36	43
*Salmonella* Typhi ATCC 6539	+ ^2^	+	+	+	+
*Salmonella* Typhimurium ATCC 14028	+	+	+	+	+
*Salmonella* Enteritidis ATCC 13076	+	+	+	+	+
*Salmonella* Gallinarum ATCC 9184	+	+	+	+	+
*Salmonella* Pullorum ATCC 9120	+	+	+	+	+
*Salmonella* Abony NCTC 6017	+	+	+	+	+
*Salmonella* Choleraesuis ATCC 10708	−	+	+		
*Salmonella* Arizonae ATCC 13314	−	−	−	−	−
*Escherichia coli* ATCC 11229	−	+	+	−	−
*Klebsiella pneumoniae* ATCC 10031	−	−	−	−	−
*Enterobacter aerogenes* ATCC 13048	−	−	−	−	−
*Campylobacter jejuni* NCTC 12662	−	−	−	−	−
*Listeria monocytogenes* ATCC 7644	−	−	−	−	−
*Enterococcus faecalis* ATCC 19433	−	−	−	−	−
*Staphylococcus aureus* ATCC 6538	−	−	−	−	−

^1^ Five phages, phiSE 7, 16, 18, 36, and 43, were isolated from chicken feces, and they belong to the *Podoviridae* family. ^2^ The phage ability to plaque on different pathogens was evaluated. (−) and (+) indicate the absence of phage plaques and the presence of phage plaques, respectively. (Copyright obtained from [20].).

**Table 2:** Efficacy of phages against *Salmonella* strains and other pathogens.

Microorganism	Bacteriophage		
	LPST18	LPST23	LPST10
*Salmonella* Typhimurium ATCC 14028	A ^1^	A	B
*Salmonella* Typhimurium ATCC 13311	C	A	C
*Salmonella* Typhimurium ST-8	A	A	A
*Salmonella* Paratyphi B CMCC 50094	A	A	B
*Salmonella* Enteritidis ATCC 13076	A	A	C
*Salmonella* Enteritidis SJTUF 10978	E	C	A
*Salmonella* Enteritidis SJTUF 10984	E	E	A
*Salmonella* Anatum ATCC 9270	D	E	D
*Salmonella* Choleraesuis ATCC 10708	E	E	D
*Escherichia coli* DH5α	E	E	E
*Escherichia coli* BL21	E	E	E
*Escherichia coli* c83715	E	E	E
*Listeria monocytogenes* ATCC 19114	E	E	E
*Vibrio parahaemolyticus* ATCC 33846	E	E	E
*Staphylococcus aureus* ATCC 29213	E	E	E
*Staphylococcus aureus* ATCC 6538	E	E	E
*Lactobacillus acidophilus* ATCC SD5221	E	E	E

^1^ A, B, C, D, and E describe the clearing throughout with a faint hazy background, substantial turbidity throughout the cleared zone, a few individual plaques, and no clearing, respectively. (Copyright obtained from [48].)

**Table 3:** Efficacy of the bacteriophage cocktail in the reduction of *Salmonella* Enteritidis in raw salmon fillets and smoked salmon slices.

Incubation (day)	Phage Cocktail Addition ^3^	Food Sample	*S.* Enteritidis (log CFU/g) ^1^		Reduction (log CFU/g)		Phage cocktail (log PFU/g) ^2^	
			18 °C	4 °C	18 °C	4 °C	18 °C	4 °C
3	−	Raw salmon fillet	7.51 ± 0.16	4.76 ± 0.20				
	+		6.76 ± 1.20	1.64 ± 0.36	0.75	3.12	6.57± 0.24	9.32 ± 0.23
6	−		6.70 ± 0.60	5.07 ± 0.17				
	+		4.13 ± 0.95	2.24 ± 0.45	2.57	2.83	7.32 ± 0.27	9.04 ± 1.82
10	−		5.90 ± 0.49	3.12 ± 0.45				
	+		2.71 ± 0.98	0.30 ± 0.43	3.19	2.82	7.80 ± 0.40	9.68 ± 0.39
3	−	Smoked salmon slice	8.23 ± 0.13	3.84 ± 0.08				
	+		6.54 ± 0.28	3.34 ± 0.18	1.69	0.5	7.30 ± 0.37	8.32 ± 0.23
6	−		8.34 ± 0.15	3.73 ± 0.26				
	+		7.32 ± 0.37	3.38 ± 0.19	1.02	0.35	6.61 ± 0.36	8.80 ± 0.07
10	−		6.96 ± 0.42	2.28 ± 0.24				
	+		5.0 ± 0.48	1.12 ± 0.32	1.96	1.16	6.27 ± 0.19	8.66 ± 0.33

^1^ The bacterial inoculums were 3.2 and 4.2 log colony-forming unit (CFU)/g, respectively, for 18 °C and 4 °C.^2^ The phage titers were 7 and 8 log10 plaque-forming unit (PFU)/g, respectively, for 18 °C and 4 °C. ^3^ (–) indicates the control samples without phage and (+) denotes the samples treated with the phage cocktail. [116].

## Appendix A

**Table A1:** Bacteriophage based sensors for detection of *Salmonella.*

Transducer	Phage Type	Phage Immobilization	Analyte	Sample	Detection Limit (CFU/mL)	Linear Range (CFU/mL)	Reference
Magnetoelastic	E2 phage	Physical adsorption	*S. typhimurium*	Tomato surface	5 × 10^2^	5 × 10^1^–5 × 10^8^	[141]
Magnetoelastic	E2 phage	Physical adsorption	*S. typhimurium*	Culture	5 × 10^3^	5 × 10^3^–5 × 10^7^	[142]
Magnetoelastic	C4-22	Physical adsorption and cysteine	*S. typhimurium*	Chicken	7.9 × 10^3^	-	[143]
Magnetoelastic	E2 phage	-	-	Tomato surface	1.5 × 10^3^	1.5 × 10^0^–1.5 × 10^6^	[144]
Magnetoelastic	E2 phage	-	-	Soil	10^2^	10^4^–10^7^	[145]
Magnetoelastic	E2 phage	-	-	Romaine lettuce	5 × 10^2^	10^1^–10^8^	[146]
Capacitive	M13 phage clone	Phage / Pty/Au electrode using glutaraldehyde linker	*Salmonella* spp.	Chicken	2 × 10^2^	2 × 10^2^–1 × 10^7^	[147]
Magnetoelastic	E2 phage	Physical adsorption	*S. typhimurium*	Fat free milk	5 × 10^3^	-	[147]
Magnetoelastic	E2 phage	Physical adsorption	*S. typhimurium*	Tomato surface	-	-	[148]
SPR	M13 Phage derived peptide	Phage /Au surface using 1-ethyl− 3-(3-dimethyl-aminopropyl) carbodiimid linker	*S. typhimurium*	Culture	10^3^	-	[149]
SPR	M13 phages	Phage /Au surface using EDC/NHS linker	*Salmonella* spp.	Culture	1.3 × 10^7^	-	[150]
Maxtek acoustic wave device	Filamentous phage	Physical adsorption	*S. typhimurium*	Culture	10^1^	10^1^–10^7^	[95]
Microcantilevers	M13 phage-derived peptides	Phage / Au surface using succinimidyl propionate linker	*Salmonella* spp.	Culture	1 × 10^6^	1 × 10^6^−1 × 10^8^	[151]
Magnetoelastic	E2 phage	Physical adsorption	*S. typhimurium*	Tomato surface	-	10^2^–10^4^	[152]
Magnetoelastic	Filamentous phage	Physical adsorption	*S. typhimurium*	Culture	10^3^	5 × 10^3^–5 × 10^6^	[93]
SPR	P22 Phage TSP	Phage /Au surface using EDC/NHS linker	*Salmonella*	Culture	10^3^	-	[153]
Bioluminescence	Felix phage or Newport phage	-	*S.* newport	Culture	10^3^	-	[154]
oluminescence	phage SJ2	-	*S.* enteritidis	Culture	10^3^	-	[155]
Magnetoelastic	E2 phage	-	*S. typhimurium*	Tomato surface	10^3^	10^3^ × 10^7^	[94]
Fluorescent	Recombinant prophage	-	*S. typhimurium*	Sea water	10	-	[86]
Magnetoelastic	E2 phage	-	*S. typhimurium*	Spinach	10^2^	-	[156]
Magnetoelastic	E2 phage	Physical adsorption	*S. typhimurium*	Eggshells	1.6 × 10^2^	1.6–1.6 × 10^7^	[157]
Magnetoelastic	Filamentous E2 phage	Physical adsorption	*S. typhimurium*	Spinach	10^2^	-	[39]

## Appendix B

**Table A2:** Applications of bacteriophage or bacteriophage-based treatments for biocontrol of *Salmonella.*

Phage Type	Phage Characteristic	Target	Related Samples	Concentration of Phage	Treatment Mode	Efficacy	References
One phage							
Virulent phage F01-E2	*Myoviridae* family, 86.2 kb dsDNA genome	*S.* Typhimurium	RTE foods including Hot dogs, cooked and sliced turkey breast, mixed seafood, chocolate milk, and egg yolk	3 × 10^8^ pfu/g	Directly adding in the samples.	At 8 °C, more than 3 log reduction resulted in no viable cells in all samples; while at 15 °C, 5 log reduction on turkey deli meat and in chocolate milk, and by 3 logs on hot dogs and in seafood. Reduction effect only obtained after 2 days in egg yolk.	[109]
Phage phSE-1	All three belong to order Caudovirales and *Siphoviridae* family	*S.* Typhimurium	In vivo test	10^7^ pfu/mL with a MOI of 100	Directly mixing.	Significant reductions of 1.8, 1.7 and 1.9 log CFU/mL were observed with phSE-1, phSE-2, and phSE-5 respectively	[158]
Phage phSE-2							
Phage phSE-5							
A phage cocktail							
A phage cocktail of UAB_Phi 20, 78, and 87)	UAB_Phi 20 and 78 belong to *Siphoviridae* family and UAB_Phi 87 is a member of *Myoviridae* family	*S.* Typhimurium and *S.* Enteritidis	Pig skin, chicken breasts, fresh eggs, and packaged lettuce	10^10^ pfu/mL for pig skin and fresh eggs, 10^9^ pfu/mL for chicken breasts and lettuce	Spraying for pig skin and fresh eggs, while agitation 5 min and 60 min for chicken breasts, and lettuce, respectively	In pig skin, >4 and 2 log/cm^2^ for *S.* Typhimurium and *S.* Enteritidis were reduced for 6 h, respectively; in chicken breasts, 2.2 and 0.9 log cfu/g for *S.* Typhimurium and *S.* Enteritidis were reduced for 7 days, respectively; in lettuce, 9 and 2.2 log cfu/g, respectively; in fresh eggs, a reduction of 0.9 log cfu/cm^2^ for 2h	[120,121]
SalmoFresh^TM^	Commercial product	*S.* Newport	Whole and fresh-cut cucumbers	10^10^ pfu/mL	Spraying	*S.* Newport was significantly lower when treated by phage at 10 ℃ on day 1 and 4.	[159]
A phage cocktail of S16 and FO1a	Both belong to the order *Caudovirales* and *Myoviridae* family	*S. enterica*, *S.* Heidelberg, *S.* Newport, and *S.* Enteritidis C, Se 13	Ground meat including beef and pork trim, and poultry including chicken and turkey thighs	10^7^ or 10^8^ pfu/mL for samples, and 10^9^ pfu/mL for vitro study	Tumbling for 2 min at 4 rpm	In vitro study, 99% were reduced for all strains; in vivo test, bacteria reductions of 1, 0.8, 1.1 and 0.9 log cfu/g were obtained in beef, pork, chicken, and turkey, respectively.	[160]
A phage cocktail of vB_SnwM_CGG4-1, 4-2, 3-1, and 3-2	vB_SnwM_CGG4-1, and 4-2 belong to *Myoviridae* family and vB_SnwM_CGG3-1, and 3-2 belong to *Siphoviridae* family	*S.* Newport	Cherry tomato	10^6^ and 10^8^ pfu/mL		In vitro study, 3 log reduction was obtained after up to 7 h incubation; in vivo test, 2 log reduction with a MOI of 10^3^ and about 4.4 log reduction was observed after 2, 3, and 4 days with a MOI of 10^5^	[161]
SalmoFresh^TM^	*Myoviridae* family	*S.* Newport, *S.* Braenderup, *S.* Typhimurium, *S.* Kentucky, and *S.* Heidelberg	Lettuce, mung bean sprouts and seeds	10^8^ pfu/mL	Spraying, immersion,	Reductions of 0.76 and 0.83 log10 CFU/g were obtained on lettuce and sprouts by spraying, respectively, while 2.43 and 2.16 log10 CFU/g by immersion.	[162]
Phage based hurdle treatment							
A cocktail of *6* phages including F01, P01, P102, P700, P800, and FL 41, combined with *Enterobacter asburiae* JX1	-	*S.* Agona, *S.* Berta, *S.* Enteritidis, *S.* Hadar, *S.* Heidelberg, *S.* Javiana, *S.* Montevideo, *S.* Muenchen, *S.* Newport, *S.* Saint Paul, and *S.* Typhimurium DT104	Sprouting mung bean and alfalfa seeds	10^6^ pfu/mL	Soaking for 20 min	In vivo, reduction of 5.7 to 6.4 log CFU/mL were obtained. In sprouting mung bean sprouts, an additive effect was observed with the combination resulted in a detectable *Salmonella* only after enrichment. For sprouting alfalfa seeds, no *Salmonella* was recovered even with enrichment	[163]
A phage cocktail of S16 and FO1a combined with UV	Both belong to the order *Caudovirales* and *Myoviridae* family	*S.* Infantis, *S.* Heidelberg, *S.* Newport, and *S.* Enteritidis C, Se 13	Ground beef	10^9^ pfu/mL	Tumbling for 2 min at 4 rpm	Approximately 1 log CFU/g reduction for bacteriophage and UV, separately, while 2 log CFU/g for combination	[160,164]
oFresh^TM^ combined with chlorinated water	*Myoviridae* family	*S.* Newport, *S.* Braenderup, *S.* Typhimurium, *S.* Kentucky, and *S.* Heidelberg	Lettuce, mung bean sprouts and seeds	10^8^ pfu/mL	Immersion 15 min for lettuce and sprouts, 1 h for mung seeds	Reductions of 3.8, and 2.7, 1.28 log CFU/g were obtained by hurdle treatment on lettuce, sprouts, and mung seeds, respectively.	[162]
